# An exploration of relocation initiatives deployed within and between nursing homes: a qualitative study

**DOI:** 10.1186/s12913-023-10505-8

**Published:** 2024-01-04

**Authors:** Damien S. E. Broekharst, Annerieke Stoop, Wilco P. Achterberg, Monique A. A. Caljouw, on behalf of the RELOCARE Consortium, Katrien G. Luijkx, Hilde Verbeek, Jan P. H. Hamers, Jos M. G. A. Schols, Bram de Boer, Judith H. J. Urlings, Mara Brouwers, Elleke G. M. Landeweer, Dika H. J. Luijendijk, Miranda C. Schreuder, Sytse U. Zuidema, Marieke Perry, Raymond T. C. M. Koopmans, Wim G. Groen

**Affiliations:** 1 Department of Public Health and Primary Care, Leiden University Medical Center, P.O. Box 9600, 2300 RC Leiden, the Netherlands; 2 University Network for the Care Sector South Holland, Leiden University Medical Center, P.O. Box 9600, 2300 RC Leiden, the Netherlands; 3 Academic Collaborative Centre Older Adults, Tranzo Scientific Centre for Care and Wellbeing, Tilburg School of Social and Behavioral Sciences, Tilburg University, P.O. Box 90153, 5000 LE Tilburg, the Netherlands

**Keywords:** Relocation initiatives, Pre-relocation initiatives, Bridging initiatives, Post-relocation initiatives, Nursing homes, Older adults

## Abstract

**Background:**

Relocations within and between nursing homes often induce stress, anxiety, and depression in residents and cause additional workload for and burnout in staff. To prevent this, many nursing homes deploy pre-transition initiatives, bridging initiatives, and post-transition initiatives to support residents and staff during the relocation process. As little is known about these initiatives, this study aims to explore the pre-relocation, bridging and post-relocation initiatives used for relocations within and between nursing homes.

**Methods:**

In seven Dutch nursing homes, eight focus groups were conducted with two to six participants (*N* = 37) who were actively involved in relocation processes in different roles (i.e., managers, healthcare professionals, support staff, client council members, residents and family). The focus groups were conducted based on a predefined topic list and lasted approximately 60 min. The transcripts were recorded, transcribed verbatim and analysed using thematic coding.

**Results:**

Nursing homes had to be inventive in developing relocation initiatives as neither shared guidelines nor knowledge exchange on this topic were available. A total of thirty-seven relocation initiatives were identified in these seven nursing homes. Nineteen pre-relocation initiatives were identified, of which eight emphasized information and engagement, three highlighted training and practice and eight stressed orientation and visualization. Seven bridging initiatives were identified, of which four emphasized coordination and continuity and three highlighted entertainment and celebration. Eleven post-relocation initiatives were identified, of which seven emphasized evaluation and troubleshooting and four highlighted change and adjustment.

**Conclusion:**

The identified relocation initiatives were developed unassisted by nursing homes, due to a lack of shared guidelines, knowledge exchange and mutual learning on this topic. Therefore, it may be expedient and more effective to develop general guidelines for relocations within and between nursing homes in collaboration with nursing homes.

**Supplementary Information:**

The online version contains supplementary material available at 10.1186/s12913-023-10505-8.

## Background

Relocation of residents within and between nursing homes occurs for a variety of reasons, ranging from changing healthcare needs to closure or renovation of nursing homes [1, 2]. Although these relocations may considerably improve the living conditions of residents and working conditions of staff over time [1–5], the actual relocation process within and between nursing homes often induces stress, anxiety, and depression in residents and causes additional workload for and burnout in staff [1–5]. In order to prevent, mitigate, or eliminate these negative consequences, nursing homes deploy relocation initiatives to support residents and staff [6, 7]. Relocation initiatives may include any set of practices, methods, protocols, policies, guidelines, or programmes designed to safeguard the coordination and continuity of healthcare as (groups of) individuals transfer between different locations or different levels of care within the same location [6, 7]. Three types of relocation initiatives can be distinguished, namely pre-relocation initiatives, bridging initiatives and post-relocation initiatives [7, 8]. Pre-relocation initiatives refer to a set of actions aimed at preparing residents and staff for their new living and working conditions [7, 8]. Bridging initiatives refer to a set of actions aimed at limiting the physical and mental strain and burden on residents and staff during the relocation to the new nursing home [7, 8]. Post-relocation initiatives refer to a set of actions aimed at facilitating the habituation of residents and staff to their new living and working conditions [7, 8]. The pre-relocation, bridging and post-relocation initiatives deployed for relocations between home and nursing home or between hospital and nursing home are widely studied [9–11]. However, little is currently known about the pre-relocation, bridging and post-relocation initiatives deployed for relocations within and between nursing homes, herewith, hindering organizational learning processes and overlooking the needs of residents and staff which subsequently introduces unnecessary unrest, uncertainty and stress among these key stakeholders [12, 13]. Therefore, this study aims to explore the pre-relocation, bridging and post-relocation initiatives that are used by nursing homes in the Netherlands for relocations within and between nursing homes [12, 13]. The results of this study present healthcare professionals, managers, administrators and policymakers in the field of nursing home care with a comprehensive overview of re-relocation, bridging and post-relocation initiatives that can be deployed for relocations within and between nursing homes.

## Methods

### Study design

In this qualitative study, semi-structured focus groups were conducted to gather information on the pre-relocation, bridging and post-relocation initiatives deployed for relocations within and between nursing homes. The focus groups encouraged collective reflection among the participants often generating broad-based agreement on different topics and providing researchers with access to knowledge, expert opinions and lived experiences [14–16].

### Data collection

Nursing homes with recent relocation experiences (< 4 years) were recruited using the partnerships within the six Dutch academic collaborative networks in care for older adults [17, 18]. In total, 22 nursing home organizations were informed of this study after which seven decided to participate. Within these seven nursing homes participants were recruited based on two important inclusion criteria, namely (1) their personal or professional involvement with the relocation process and (2) their ability and availability to share experiences with the researchers. The most important challenge in the recruitment process revolved around the contamination risk associated with the COVID-19 pandemic, which could be mitigated by emphasizing close adherence to relevant rules and regulations during focus groups. Eventually, eight focus groups with two to six participants (*N* = 37) were conducted in these seven nursing homes across the Southern and Western regions of the Netherlands. A predefined topic list (Supplementary file 1) was formulated for this study including questions on (1) the pre-relocation, bridging and post-relocation initiatives deployed to facilitate relocations (e.g., What initiatives were used to prepare for the relocation of this nursing home?), (2) the processes and tools (e.g., policy documents, checklists, communication plans) used to organize relocations (e.g., What tools have been used to support the relocation of this nursing home?), and (3) the barriers and facilitators experienced by relevant stakeholders during relocations (e.g., Which factors impeded or facilitated the relocation of this nursing home?). A closing questions on tips and tricks regarding the use of relocation initiatives was also asked (e.g., Which tips regarding the use of relocation initiatives would you like to convey to other nursing homes?). All participants provided the researchers with their written consent before the start of the focus groups. Each focus group lasted approximately 60 min, was audio recorded and transcribed verbatim. After analysing the data from these eight focus groups, the researchers concluded that data saturation was achieved. Data saturation marks the point in a research process where sufficient data has been gathered in order to draw reliable conclusions, and any additional data collection will not generate new insights [19]. Transcripts were pseudonymized by excluding personal and organizational details from the transcripts.

### Data analysis

The transcripts were examined using framework analysis [20–22]. A set of predetermined and overarching codes was developed by the researchers (i.e., deductive approach) [20–22]. Based on the content of the transcripts, the predetermined codes were further explicated in multiple subcodes and expanded if new codes emerged (i.e., inductive approach) [20–22]. This generated a final version of the analytical framework that was applied to the transcripts [20–22]. This analytical framework initially consisted of three main themes (i.e., preparation, bridging, aftercare) which were each divided into three subthemes (i.e., relocation initiatives, relocation tools, barriers and facilitators). One subtheme was added to the initial framework due to advancing insight (i.e., COVID-19 effects). The initial analytical framework was based on a prior scoping review [13], other relevant research [7, 8, 12] and a review of relevant documents (e.g., policy documents, relocation protocols) collected from participating nursing homes. The framework analysis was conducted by two independent researchers who had a background in healthcare research and also had considerable experience with qualitative methods. Possible differences of opinion were discussed until consensus was reached and if no consensus could be achieved, a third independent researcher was available for consultation [20–22]. The analysis was performed using the software package Atlas.ti 22 [23].

### Reliability and validity

The reliability and validity of this study has been optimized by including multiple perspectives, different types of nursing homes and different ways of relocating as this could improve generalizability. The reliability and validity of this study was also optimized by analyzing data with two experienced researchers as this may reduce bias.

## Results

### Population characteristics

The nursing homes included in this study were of different capacities. In total, three smaller nursing homes (< 50 residents), two average-sized nursing homes (50–150 residents) and two larger nursing homes (> 150 residents) were included. The types of relocations examined in this study also differed. Four nursing homes relocated to a new residence due to closure of the existing location, two nursing homes relocated to a temporary location and back due to renovation, and in one nursing home changing healthcare needs and preferences resulted in a considerable number of internal individual relocations. The participants in this study were all actively involved in the relocation process in different roles. In total, ten managers (i.e., location managers, regional managers), ten healthcare professionals (i.e., nurses, psychologists, elderly care physicians), nine support staff (i.e., quality officers, facility officers, property maintenance employees), four client council members (i.e., local council members, general council members) and four residents and family members (i.e., daughters, husbands) were included.

### Relocation initiatives

Respondents indicated that nursing homes had to be inventive, creative and innovative in developing and implementing different pre-relocation, bridging and post-relocation initiatives for relocations within and between nursing homes, as neither shared guidelines nor knowledge exchange on this topic were available to them. Respondents reported thirty-seven relocation initiatives, of which nineteen were pre-relocation initiatives, seven were bridging initiatives and eleven were post-relocation initiatives. The pre-relocation, bridging and post-relocation initiatives identified in this study are summarized in Table 1.

**Table 1:** Initiatives used for relocations within and between nursing homes

Phase of initiative	Theme of initiative	Type of initiative	Description of initiative	Mentions
Pre-relocation initiatives	Information and engagement	Workgroups	Group of staff members that develops, implements and executes activities regarding the impending relocation	5
		Information meetings	Meeting in which information is shared with residents, family and staff about the impending relocation	6
		Newsletters	Periodical bulletin in which residents, family and staff are informed and updated on the impending relocation	5
		Walk-in consultations	Open consultation in which residents, family and staff can air concerns and ask questions about the impending relocation	2
		Press releases	Public message in which community members are informed and updated on the impending relocation	2
		Community engagement	Forum or activity in which community members are involved in or informed about the impending relocation	4
		Room and interior choice	Shared decision-making with residents and family on basic room and interior requirements desired after the impending relocation	5
		Personal interviews	Individual meeting with residents and family about healthcare needs of the residents regarding the impending relocation	6
	Training and practice	Training	Courses on the use of domotics, hospitality, cooking and dementia that prepare staff for the impending relocation	4
		Technology testing days	Days designated to test and practice with the new technology and domotics used at the new residence	1
		Simulation days	Days designated for staff to practice with and adjust to healthcare delivery at the new residence	1
	Orientation and visualization	Illustrations	Representation that provides residents, family and staff with a detailed impression of the new residence	3
		Floor plans	Scale diagram informing residents, family and staff on room arrangement and room interior at the new residence	3
		Instruction videos	Video that teaches residents, family and staff how to conduct certain practices and acts related to the impending relocation	4
		Vlogs	Video diaries that members of staff often post in which they record their thoughts and experiences on the impending relocation	1
		Mood boards	Artistic arrangements of images, materials and text projecting the design and style of the new residence	2
		Mock-up rooms	Full-size representations of rooms at the new residence built to evaluate the proposed design of the new residence	2
		Viewing days	Day designated for visits by residents, family and staff to get an impression of the construction of the new residence	7
		Guided tours	Tour for residents, family and staff with a guide who shows them around and informs them on the new residence	3
Bridging initiatives	Coordination and continuity	Relocation director	Professional who is in charge of the relocation and supervises the activities of other staff during the relocation	5
		Buddy system	Arrangement that pairs staff with residents giving them responsibility over their safety and wellbeing during the relocation	2
		Briefings	Meeting at which instructions are provided to residents, staff and family before and during the relocation	1
		Practical assistance	Support with packing, moving, cleaning and furnishing provided to residents by staff and family during the relocation	4
	Entertainment and celebration	Recreation	Leisure activities including performances, games, gifts and food used to alleviate stress among residents during the relocation	2
		Festive farewell	Celebratory event organized in order to suitably say goodbye to the old residence just before or during the relocation	3
		Festive welcome	Celebratory event organized in order to pleasantly open the new residence during of just after the relocation	3
Post-relocation initiatives	Evaluation and troubleshooting	Scrum meetings	Meeting in which staff develops and implements work processes and procedures to be used after the relocation	1
		Lean boards	Visual tool that helps teams manage their continuous improvement efforts after the relocation	1
		Signalling systems	System used for registering and signalling (technical) malfunctions experienced after the relocation	2
		Satisfaction questionnaires	Survey that monitors the experiences of staff, residents and family regarding the relocation and new residence	2
		Auxiliary staff	Healthcare professionals who support the core workforce of the nursing home just after the relocation	2
		Complaints procedure	Process that allows complaints about the relocation and new residence to be presented, recorded and dealt with effectively	1
		Evaluation meetings	Staff meeting in which different aspects of the relocation and new residence are systematically assessed	5
	Change and adjustment	Job coach	Professional who helps staff adjust to the working conditions after relocation to the new residence	1
		Culture trainer	Professional who helps staff adapt to the organizational norms and values after relocation to the new residence	1
		Lean coach	Professional who helps staff to craft new work processes and procedures to be used after relocation to the new residence	1
		Resting period	Period after the relocation in which rest and acclimatization is promoted and prioritized among residents and staff	3

### Pre-relocation initiatives

Respondents reported three types of pre-relocation initiatives, namely initiatives concerning 1) information and engagement, 2) training and practice, and 3) orientation and visualization.

#### Information and engagement

Respondents stated that pre-relocation initiatives concerning information and engagement (i.e., workgroups, information meetings, newsletters, walk-in consultations, press releases, community engagement, room and interior choice, personal interviews) are deployed in order to establish support, mitigate anxiety, prevent distrust and identify preferences with regard to the impending relocation to the new residence. One respondent mentioned:

*"Providing information is a very important one, both for our frail elderly and for the relatives of our residents, […]. Because practice shows that this generates a lot of questions, so information is very important, as concrete as possible in terms of dates and things like that." (Facility officer)*

Respondents stated that of the above-mentioned pre-relocation initiatives, workgroups, information meetings and newsletters were often deployed by nursing homes and were experienced as valuable, because (mass) communication with and engagement of residents, family and staff prevents anxiety and unrest. They also indicated that personal interviews as well as room and interior choice (e.g., selecting chairs, choosing dishes) were experienced as useful, but that these types of initiatives could only be utilized to a certain extent, as accommodating everyone’s personal preferences proved to be difficult. Respondents stated that these pre-location initiatives could all be used for group relocation, while interior and room choice as well as personal interviews were also used for individual relocations.

#### Training and practice

Respondents indicated that pre-relocation initiatives concerning training and practice (i.e., training, technology testing days, simulation days) are deployed in order to enhance preparation, safeguard quality and ensure safety with regard to the impending relocation to the new residence. One respondent stated:

*"But, of course, we appointed a number of key users who were initially fully trained and informed on how to use those home automation systems. And these key users, they passed that on to the others." (Team manager)*

Respondents reported that of the above-mentioned pre-relocation initiatives, trainings were frequently deployed by nursing homes and were experienced as useful, because education of staff facilitates smooth transition to and prevents possible problems at the new residence. They also stated that more simulation days would have been much appreciated, but that this was often not feasible due to safety risks posed by unfinished construction and faulty technology at the new residence. Respondents indicated that these pre-location initiatives could all be used for group relocation, while they were not used as much for individual relocations.

#### Orientation and visualization

Respondents suggested that pre-relocation initiatives concerning orientation and visualization (i.e., illustrations, floor plans, instructional videos, vlogs, mood boards, mock-up rooms, viewing days, guided tours) are deployed in order to improve navigation and promote habituation after the impending relocation to the new residence. One respondent mentioned:

*"We organized viewing days, and they could go and look at apartment. This is the apartment. We had made a little sketch. You can put TV here or there is room for a cabinet there, for example. We will get you a fridge. So people were just totally prepared." (Location manager)*

Respondents stated that of the above-mentioned pre-relocation initiatives, illustrations, floor plans and instruction videos were often deployed by nursing homes and were experienced as rather helpful, because these are user-friendly, accessible and effective ways to provide residents, family and staff with an impression of the new residence. They also mentioned that viewing days or guided tours were experienced as useful, but that these were sometimes difficult to plan and conduct due to the COVID-19 pandemic regulations (e.g., social distancing, group size restrictions) that existed at the time. Respondents indicated that these pre-location initiatives could all be used for group relocation, while viewing days and guided tours were also used for individual relocations.

### Bridging initiatives

Respondents indicated two types of bridging initiatives, namely initiatives concerning 1) coordination and continuity, and 2) entertainment and celebration.

#### Coordination and continuity

Respondents stated that bridging initiatives concerning coordination and continuity (i.e., relocation director, buddy system, briefings, practical assistance) are deployed in order to manage complexity, streamline processes and provide stability during the relocation to the new residence. One respondent stated:

*"We are used to conferring with each other about many things. But for a move like this, it needs to be very clear who is in charge. Who decides, and if I say left, we all go left - not right. And not spend hours arguing with each other." (Regional manager)*

Respondents indicated that of the above-mentioned bridging initiatives, relocation directors were frequently deployed by nursing homes and were experienced as essential, because their presence created a focal point for swift decision-making and problem-solving, which prevents uncertainty and dissonance during the relocation. They further stated that implementing buddy systems would certainly be valued, but that these were not always feasible due to their labour-intensive nature as well as the lack of staff and volunteers. Respondents indicated that these bridging initiatives could all be used for group relocation, while practical assistance was also used for individual relocations.

#### Entertainment and celebration

Respondents stated that bridging initiatives concerning entertainment and celebration (i.e., recreation, festive welcome, festive farewell) are deployed in order to alleviate stress, provide distraction, promote unity and commemorate successes during the relocation to the new residence. One respondent mentioned:

*"Saying goodbye is also part of the whole thing, of course. Because there are many sentiments, certainly among employees, but also among residents. There are people who have worked in that building for thirty, forty years, so the moment you leave there is significant, and you have to acknowledge that. So one of the things we said was, we will say goodbye in an appropriate manner and we organized a big party there." (Regional manager)*

Respondents reported that of the above-mentioned bridging initiatives, festive farewells and festive welcomes were relatively often deployed by nursing homes and were experienced as a pleasant distraction, because these initiatives provided residents, family and staff with an opportunity to celebrate an important milestone in the relocation process. These initiatives were sometimes difficult to organize due to the COVID-19 pandemic regulations (e.g., social distancing, group size restrictions) that existed at the time. Respondents argued that these bridging initiatives could all be used for group relocation, while they were not used as much for individual relocations.

### Post-relocation initiatives

Respondents described two types of post-relocation initiatives, namely initiatives concerning 1) evaluation and troubleshooting, and 2) change and adjustment.

#### Evaluation and troubleshooting

Respondents indicated that post-relocation initiatives concerning evaluation and troubleshooting (i.e., scrum meetings, lean boards, signalling systems, satisfaction questionnaires, auxiliary staff, complaints procedure, evaluation meetings) are deployed in order to recognize complications, assess risks and resolve problems following the relocation to the new residence. One respondent stated:

*"Initially, there were notebooks everywhere for people to write in, to report defects, points of interest, and [the project leader] in particular made the rounds many times to collect all that." (Nurse)*

Respondents reported that of the above-mentioned post-relocation initiatives, evaluation meetings were most frequently deployed by nursing homes and were experienced as highly necessary, because these meetings provide the organizational feedback loop and learning capacity necessary for maintaining quality of care at the new residence. They suggested that, if available, auxiliary staff was readily deployed to ensure the safety of residents by monitoring entrances and exits, technical malfunctions and the COVID-19 pandemic regulations (e.g., social distancing, group size restrictions). Respondents stated that these post-relocation initiatives could all be used for group relocation, while complaints procedures and evaluation meetings were also used for individual relocations.

#### Change and adjustment

Respondents indicated that post-relocation initiatives concerning change and adjustment (i.e., job coaches, culture trainers, lean coaches, resting period) are deployed in order to transform culture, increase efficiency and promote adaptation following the relocation to the new residence. One respondent mentioned:

*"It is still about vision and behaviour. And we have to - I think that we sometimes are not aware enough that it requires behavioural change. And that's why I think we need to spend most of our time on stimulating people to make those changes." (Location manager)*

Respondents stated that of the above-mentioned post-relocation initiatives, resting periods were often introduced by nursing homes and were experienced as beneficial, because freezing new organizational changes or additional nursing home admissions for a while may help residents, family and staff adjust to the new residence. They further suggested that if adjustment and change become a problem, job coaches and culture trainers might be particularly useful as they could actively engage with residents, family and staff to help them adapt to the new residence. Respondents reported that these post-relocation initiatives could all be used for group relocation, while they were not used as much for individual relocations.

## Discussion

This study aimed to explore the pre-relocation, bridging and post-relocation initiatives deployed by the participating nursing homes for relocations within and between nursing homes. Nineteen pre-relocation initiatives were identified, of which eight emphasized information and engagement, three focussed on training and practice, and eight emphasized orientation and visualization. Seven bridging initiatives were identified, of which four emphasized coordination and continuity and three focussed on entertainment and celebration. Eleven post-relocation initiatives were identified, of which seven emphasized evaluation and troubleshooting and four focussed on change and adjustment.

The pre-relocation initiatives focussing on information and engagement, training and practice as well as orientation and visualization found in this study made up the majority of the initiatives and have also been reported in earlier research [8, 24–28]. However, as the specific pre-relocation initiatives regarding engagement found in this study seem limited to relatively trivial topics and often do not include the opinions of family members [13], they may be considered somewhat superficial (e.g., selecting chairs, choosing dishes) in comparison to the pre-relocation initiatives reported in scientific literature (e.g., co-designing nursing home services [29], co-creating health technology development [30, 31], participatory care planning [32, 33]). The bridging initiatives and post-relocation initiatives that stress coordination and continuity, entertainment and celebration, evaluation and troubleshooting as well as change and adjustment, were less prevalent in this study and are also much less discussed in existing research [8, 24–28]. Given these findings, one might suggest that the emphasis on pre-relocation initiatives results from a human tendency to focus on the tasks that are most straightforward, immediate and instantaneous as scientific literature indicates [34]. Nevertheless, due to this particular focus, limited attention has been directed to the development and implementation of bridging initiatives and post-relocation initiatives. Post-relocation initiatives in particular are overlooked, as only a few nursing homes deployed post-relocation initiatives with a focus on, for instance, organizational change and adjustment of residents, family and staff. This is remarkable, as previous research suggests that large relocation processes in healthcare are often accompanied by new ways of working and innovative perspectives on healthcare delivery [35, 36]. Based on this observation one might suggest that nursing homes should be more attentive to the development and implementation of post-relocation initiatives during relocations within and between nursing homes, in particular related to organizational change and adjustment. These post-relocation initiatives regarding organizational change and adjustment cultivate willingness to change among residents, family and staff making them more susceptible to, for instance, new modes of healthcare delivery and adoption of healthcare technology which could considerably improve quality of life and care [35, 36]. In addition, it has to be mentioned that organizational change and adjustment should not necessarily be considered a post-relocation initiative but rather a continuous thread that runs through all relocation phases as research shows that organizational change and adjustment are complex processes that take a long time and need to be systematically, gradually and incrementally developed and implemented [35, 36].

Finally, it should be noted that the development and implementation of pre-relocation, bridging and post-relocation initiatives as found in this study are also impacted by the COVID-19 pandemic. The COVID-19 pandemic regulations (e.g., social distancing, group size restrictions) impeded, for instance, certain relocation initiatives emphasizing information and engagement as well as orientation and visualization, which was confirmed by other studies [37–39]. In addition, this study suggests that the pandemic circumstances shifted the focus from relocation initiatives with physical and collective components to relocation initiatives with more virtual and individual aspects. Based on this reflection one might suggest that nursing homes should always be aware of and prepare for possible pandemic outbreaks or other crisis situations during relocations within and between nursing homes.

### Limitations and strengths

This study has strengths and limitations that should be considered in the interpretation of its findings. One strength of this study is that all relevant stakeholders and all phases of relocation are taken into account, generating comprehensive and complete findings. Another strength is the relatively large sample (*N* = 37), which resulted in data saturation and increased generalizability. A limitation of this study might be that the focus groups included participants with different levels of authority, which may have caused restraint, socially desirable responses and a tendency to avoid conflict among participants. Another limitation of this study might be that relatively few residents and family members were included in the focus groups, possibly resulting in underrepresentation.

### Practical implications

The findings of this study have practical implications for residents, family and staff involved in the relocation process. The findings imply that nursing homes could be more attentive to organizing a comprehensive, dialectical and reflective post-relocation phase than is currently the case. This could be achieved by, for example, deploying the mentioned post-relocation initiatives more frequently and structurally. The findings also imply that the gradual and incremental process of organizational change and adjustment may be started earlier than is usually the case. Nursing homes can achieve this by deploying the mentioned post-relocation initiatives related to change and adjustment earlier in the relocation process.

### Future research

An important direction for future research relates to the development of an evidence-based comprehensive relocation initiative that maps out a feasible planning for the relocation process and its phases, specifies important milestones for each relocation phase, highlights processes that need to be organized and executed in each relocation phase, and recommends applicable initiatives that may be deployed during each relocation phase. This comprehensive relocation initiative should also be adaptable to different types of relocations (e.g., individual, group, within, between, renovation, closure) and different types of healthcare needs (e.g., psychogeriatric, somatic, rehabilitation). It could be particularly beneficial to develop this comprehensive relocation initiative in collaboration with individuals working or living in nursing homes (e.g., managers, healthcare professionals, support staff, client council members, residents and family) as they possess a wealth of experience and knowledge on this issue.

## Conclusion

Given the many different relocation initiatives mentioned in this study, it became evident that nursing homes were inventive, creative and innovative in developing and implementing different pre-relocation, bridging and post-relocation initiatives for relocations within and between nursing homes. However, it also became apparent that each of the seven nursing homes had to develop and implement their own initiatives unassisted, as neither shared guidelines nor knowledge exchange on this topic were available to them. Therefore, It may be beneficial to facilitate and encourage a mutual exchange of knowledge on this topic among nursing homes and deploy this collective knowledge to develop and implement comprehensive guidelines for relocations within and between nursing homes.

### Supplementary Information

**Additional file 1.**

## Data Availability

The transcripts analysed during the current study are available from the corresponding author on reasonable request.
