# Supplementary material for: An exploration of relocation initiatives deployed within and between nursing homes: a qualitative study
Source: BMC Health Serv Res. 2024 Jan 4;24:22. doi: 10.1186/s12913-023-10505-8 (PMC10768348; doi:10.1186/s12913-023-10505-8)
Supplement: Supplementary file 1 — Additional file 1. [file 12913_2023_10505_MOESM1_ESM.docx]

**Topic list**

*Opening*

- What are your names, occupations and organisations?
- Which relocation processes did recently occur in your organisation?
- What role did you have in this relocation process?

*Relocation initiatives*

- What initiatives were used to prepare for the relocation of this nursing home?
- How did you experience these initiatives?
- What initiatives were used during the actual relocation day of this nursing home?
- How did you experience these initiatives?
- What initiatives were used in the aftermath of the relocation of this nursing home?
- How did you experience these initiatives?

*Relocation tools*

- What tools have been used to support the relocation of this nursing home?
- How did you experience these tools?

*Barriers and facilitators*

- Which factors impeded or facilitated the relocation of this nursing home?
- How can these barriers be overcome and these facilitators be utilized?

*Closing*

- Which tips regarding the use of relocation initiatives would you like to convey to other nursing homes?
- Would you like to be informed about the results of this study?
- Do you have any questions for the researchers?
